# The impact of containment measures and monetary and fiscal responses on US financial markets during the COVID-19 pandemic^☆^

**DOI:** 10.1016/j.heliyon.2023.e15422

**Published:** 2023-04-12

**Authors:** Emmanuel Joel Aikins Abakah, Guglielmo Maria Caporale, Luis Alberiko Gil-Alana

**Affiliations:** a University of Ghana Business School, Accra, Ghana; b Brunel University London, UK; c University of Navarra, Spain

**Keywords:** Covid-19, Policy responses and announcements, Containment measures, US financial Markets, Stocks, Bonds, Islamic stocks, Green bonds, JEL Classifications: C22, C32, G15

## Abstract

This paper analyses the effects of containment measures and monetary and fiscal responses on US financial markets during the Covid-19 pandemic. More specifically, it applies fractional integration methods to analyse their impact on the daily S&P500, the US Treasury Bond Index (USTB), the S&P Green Bond Index (GREEN) and the Dow Jones (DJ) Islamic World Market Index (ISLAM) over the period 1/01/2020–10/03/2021. The results suggest that all four indices are highly persistent and exhibit orders of integration close to 1. A small degree of mean reversion is observed only for the S&P500 under the assumption of white noise errors and USTB with autocorrelated errors; therefore, market efficiency appears to hold in most cases. The mortality rate, surprisingly, seems to have affected stock and bond prices positively with autocorrelated errors. As for the policy responses, both the containment and fiscal measures had a rather limited impact, whilst there were significant announcement effects which lifted markets, especially in the case of monetary announcements. There is also evidence of a significant, positive response to changes in the effective Federal funds rate, which suggests that the financial industry, mainly benefiting from interest rises, plays a dominant role.

## 1 Introduction

The Covid-19 pandemic has had devastating effects on the world economy which exceed those of the 2007-8 global financial crisis (GFC) or indeed other pandemics or crises [1,2], for instance, the fall in crude oil price has been the largest since the Gulf war [3]. Further economic consequences are expected to become apparent over time [[4], [5], [6]]. According to the Worldometer Data Tracker (WDT), the number of global Covid-19 cases as of May 25, 2021 had reached 167, 986, 053 with about 3.4 million deaths and a total of over 149 million recovery cases; at the time, the US had the highest number of recorded cases in the world (over 33 million with 604,385 deaths and over 27 million recoveries). Efforts to reduce the spread of the virus by imposing lockdowns and temporarily stopping various economic activities posed solvency risks for firms. The low global demand, supply and productivity affected output. Early estimates predicted that global GDP growth would drop from 3.0 to 2.4% during 2020, which represented a loss of about 3.5 trillion US dollars [7].

In the case of financial markets, the negative impact has been greater than at the time of the Spanish Flu [8], and the huge increase in systemic risk has virtually eliminated safe havens for investors [9]. The types of financial markets examined by previous studies include international and domestic equity markets [4,[10], [11], [12], [13], [14]], commodity markets such as gold and oil [3,15,16], alternative assets class including cryptocurrencies [[17], [18], [19]], the debt market [[20], [21], [22]] and mutual funds [23].

Governments worldwide have had to adopt wide-ranging policy measures in response to the pandemic [24,25]. These include containment measures restricting social interaction (such as workplace, schools and restaurants closures) as well as both domestic and international travel; monetary measures such as lowering policy rates (e.g., Australia, Argentina, Brazil, Chile, Canada, Mexico, India and UK), expanding quantitative easing (e.g., US), introducing new targeted long-term refinancing operations (e.g., Eurozone), lowering the reserve requirement ratio (e.g., Brazil, China); fiscal measures such as adopting income support and debt relief schemes (US etc.). The impact of these policy actions specifically on financial markets as opposed to the economy as a whole has only been analysed by a handful of studies. In particular [26], examined the effect of policy responses on global stock market liquidity and found that workplace and school closures deteriorate liquidity in emerging markets, while information campaigns on the virus boost trading activity [27]. concluded that the pandemic has significantly weakened the transmission of monetary policy to financial markets [10]. reported that stock markets were negatively impacted by government announcements of restrictions, whilst policies imposing quarantining and testing had a positive effect [28]. found that stock markets in the G7 were positively affected by economic support and travel bans [29]. provided evidence that policy interventions during the pandemic in some cases increased market uncertainty.

Policy responses can affect returns on financial instruments through a number of channels. First, the closure of workplaces and schools, which are described as the “infrastructure channel”, can have an impact on the decision-making processes of firms; in addition, investors may not be able to conduct transactions when financial institutions or firms are physically closed [30,31]. Second, policy measures can signal possible future changes in economic activity and thus lead to a restructuring of portfolio strategies – this is known as the “portfolio channel”. For example, if markets conditions deteriorate, investors may decide not to allocate money to risky assets such as stocks. Further, workplace closures can result in the expectation of lower future household income [30] and thus increase the risk premium [32]. Third, psychological and behavioural factors can influence investors. For instance, market participants might monitor their portfolios more closely during more volatile market conditions and in the wake of continuous announcements of government restrictions may simply want to “put their head in the sand” instead of investing, which is known as the “ostrich effect” [33,34].

The present study considers the impact on a wide range of US asset prices (specifically, standard stock and bond prices, and also Islamic stock and green bond prices) of Covid mortality rates as well as containment, fiscal and monetary responses and announcements, and thus it takes into account the effects of both the pandemic itself and the policy measures adopted in response to it using a comprehensive framework. In contrast to previous studies, the modelling approach is based on the concept of fractional integration, which is much more general than standard methods based on the I (0)/I (1) dichotomy since it allows for fractional values of the integration parameter d and therefore for a much wider range of possible stochastic behaviours of the series under examination. The main objective of the present study is to analyse the effects of the mortality rates resulting from the Covid-19 pandemic as well as of fiscal and monetary responses on the behaviour of US stock prices. In particular, the fractional integration methods used enable us to investigate if these health and policy shocks have had transitory, long lasting or even permanent effects on the dynamics of the series of interest. Note that the chosen econometric approach is more general in comparison to that used on earlier studies which were based on the classical dichotomy between I (0) and I (1) processes; by contrast, our framework includes various types of stochastic processes such as nonstationary but mean-reverting ones occurring when the order of integration is in the range [0.5, 1). Obtaining comprehensive, reliable evidence for the US using these methods is an important contribution to the literature.

Other studies published in Heliyon Business and Economics have analysed the relationship between the Covid-19 pandemic and stock markets. For instance Ref. [35], found that during the Covid-19 period volatility spillovers and contagion across and within Islamic and/or G7 markets increased [36]. reported that Covid-19 cases had a significant long-term impact on the exchange rate returns and stock markets returns of the fifteen most affected countries [37]. concluded that responses from stock markets to the Covid-19 pandemic changed over time in the Asia-Pacific region and that adopting pandemic control measures helped reduce market volatility at the country and region levels [38]. found that Covid-19 vaccination had a positive impact on the stock markets of developing countries and a negative one on those of developed countries [39]. provided evidence that there was no significant impact of the Covid-19 pandemic on the degree of persistence of the European stock market indices, though their volatility persistence decreased. The present paper differs from the above mentioned ones and contributes to the Heliyon debate in three respects, namely by (i) focusing exclusively on the US but analysing different types of US stocks and bonds, (ii) examining at the same time the impact of both Covid-19 health measures and other policy measures, and (iii) using a methodology (specifically, fractional integration) that encompasses a wider range of dynamic behaviours than previously considered. Note that the only other contribution concerning Covid-19 and stock markets which uses fractional integration techniques (i.e. [39]) has a European focus and only assesses the impact of the pandemic itself on one specific property of stock prices (i.e., their persistence) rather than considering a whole range of factors that drove stock and bond prices in the US during that period as the current study does.

The layout of the paper is as follows: Section 2 outlines the econometric framework; Section 3 describes the data and presents the main empirical findings; Section 4 discusses some policy implications of the results obtained, while Section 5 offers some concluding remarks.

## 2 Econometric framework

We consider the following regression model:

(1) $$\begin{array}{cc} y\left(t\right)=\beta^{T}\;z\left(t\right)+x\left(t\right)\text{;} & {\left(1-L\right)}^{d}x\left(t\right)=u\left(t\right)\text{.} \end{array}$$

where *y(t)* is the observed time series representing each of the stock market indices in turn, namely the S&P 500 Composite Index (SP500), the S&P Treasury Bond Index (USTB), DJ Islamic Market World Index (ISLAM) and S&P Green Bond Index (GREEN); *β* is a (8.×1) vector of unknown parameters including a constant and seven other coefficients; *z(t)* = (1, CHI(t), ISP(t), DRP(t), EFFR(t), MMFPM(t), FP(t), DR(t))^T^ is the vector including the regressors, where CHI stands for the Containment Health Index, ISP for Income Support Policy, DRP for Debt-Relief Policy, EFFR for the Effective Federal Funds Rate, MMFPM and FP are two dummies corresponding to policy announcements concerning (i) Monetary and Macro-Financial Policy Measures and (ii) Fiscal Policy, and DR for the Mortality Rate per 100,000 people**;** L is the lag operator, i.e., L^k^x(t) = x (*t*-k); *x(t)* assumed to be an integrated of order d or *I(d)* process where the differencing parameter *d* is also to be estimated from the data; finally *u(t)* is an *I(0)* process, which is assumed in turn to be a white noise process or to be weakly autocorrelated. Note that the second equation in (1) implies that *x(t)* is integrated of order *d* and thus if *d > 0* the series display long memory, which imply that they are highly dependent, with higher values of d indicating higher dependence between the observations, even if they are far apart in time.

The estimation is carried out for the d-differenced regression following the approach developed in Robinson [40]; a simple version of this procedure tests the null hypothesis **as specified in** equation (2) **below:**

(2) $$H_{0}:d=d_{o},$$

in (1) for any real value *d*_*o*_. Thus, under the null hypothesis H_o_ (2), the two equalities in equation (1) can be expressed as in equation (3) below:

(3) $$\tilde{y}\left(t\right)=\beta^{T}\tilde{z}\left(t\right)+u\left(t\right)$$

where $\tilde{y}\left(t\right)={\left(1-L\right)}^{d_{o}}y\left(t\right)$ and $\tilde{z}\left(t\right)={\left(1-L\right)}^{d_{o}}z\left(t\right)\text{,}$ and noting that *u(t)* is *I(0)* by construction, the estimation of *β* can be carried out using OLS (GLS) (see, e.g. Ref. [41] for a full description of this procedure).

## 3 Empirical analysis

**Fig. 1:**
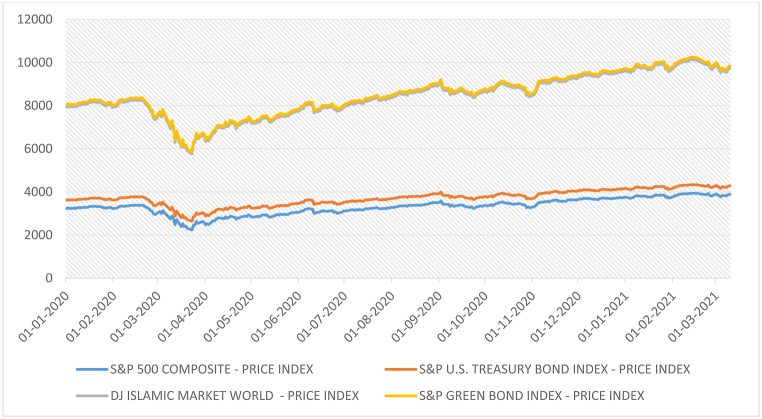
Stock and bond indices.

The four series examined are the daily log-returns of S&P 500 Index, US Treasury Bond Index, S&P Green Bond Index and Dow Jones (DJ) Islamic World Market Index obtained from Datastream from January 1, 2020 to March 10, 2021. Fig. 1 contains plots of all four of them. Their evolution over time is rather similar, namely they fall sharply in the first quarter of 2020, when the impact of the pandemic was first felt, reaching their bottom around April–May 2020, when the US witnessed a significant increase in the number of Covid-19 cases and tighter social interaction restrictions were imposed; then they resumed their growth, even exceeding their values at the beginning of the sample in the case of the two non-conventional (Islamic and green) indices.

**Fig. 2:**
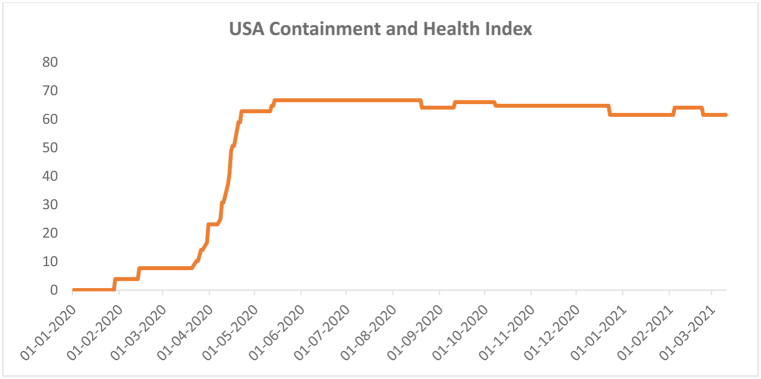
Containment and health index.

The Covid-19 policy response measures have been taken from the Oxford Coronavirus Government Response Tracker (https://ourworldindata.org/policy-responses-covid.com). The Containment and Health Index is a composite measure based on: workplace closures, school closures, public events cancellations, public gatherings restrictions, public transport closures, stay-at-home restrictions, public campaigns restrictions, internal movement restrictions, restrictions on international travels, testing policy, magnitude of contact tracing, covering of face and vaccine policy. The index on any given day is calculated as the mean score of the thirteen metrics, each taking a value between 0 and 100. A higher score indicates a stricter response (i.e. 100 = strictest response). Fig. 2 displays a plot of this series; the adoption of stricter policies around April–May 2020 is immediately apparent.

**Fig. 3:**
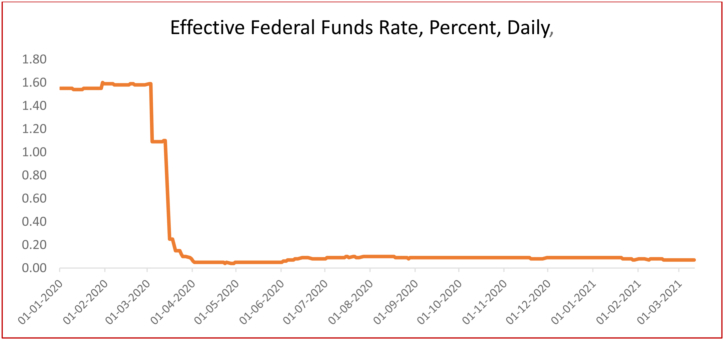
Daily effective federal funds rate.

The fiscal policy response variables include: income support, which provides information about the extent to which the US government has covered salaries or provided universal basic income, direct cash payments, or similar, to people who lost their jobs or could not work; debt or contract relief, which indicates whether the US government froze loan repayments and other types of utility payments, banned evictions etc. During the pandemic. Finally, the effective Federal Funds rate is included to account for monetary policy responses. This variable is plotted in Fig. 3; it can be seen that this rate was cut sharply in March–April 2020 and has then been kept at the new low level.

We also construct shift dummies corresponding to key dates when the US government made monetary policy and fiscal policy announcements. In the case of the former (MMFPM), the chosen date is March 15, 2020, when the Federal Funds rate was lowered by 150bp to 0–0.25bp. As for fiscal announcements (FP), the following dates were selected: December 28, 2019, when President Trump signed a US $ 868bn (about 4.1% of GDP) coronavirus relief and government funding bill as part of the Consolidated Appropriations Act of 2021; August 8, 2020, when he issued executive orders, mostly to address the expiration of certain Coronavirus reliefs provided by previous legislation; March 11, 2021, when the House of Representatives approved the American Rescue Plan, which provides another round of coronavirus relief with an estimated cost of $1,844bn (about 8.8% of 2020 GDP).

**Fig. 4:**
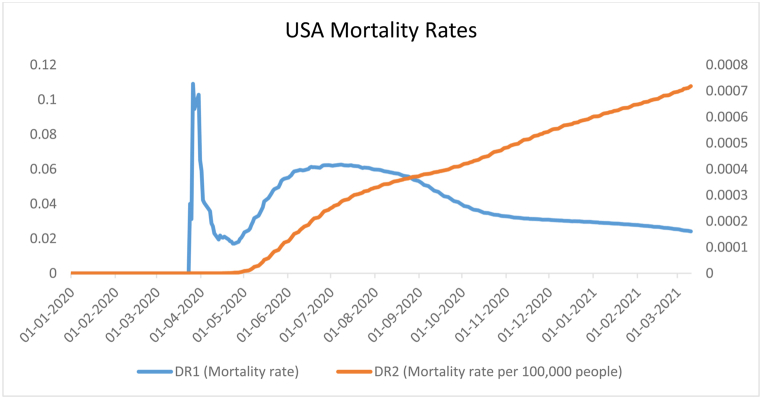
Plot of US mortality rates during the COVID-19 period.

**Fig. 5:**
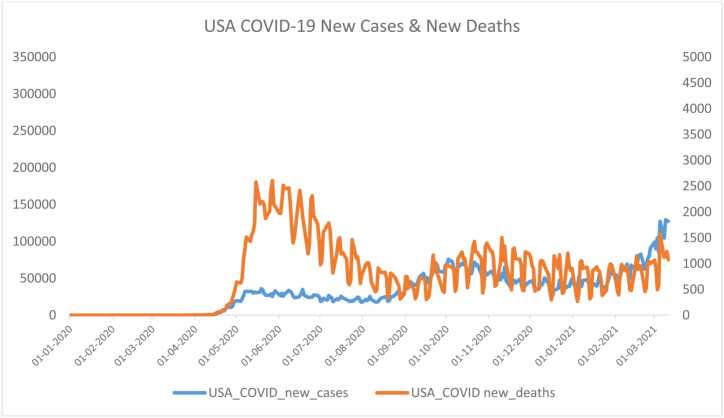
US COVID-19 new cases & new deaths.

Finally, following [42], the direct impact of the pandemic is taken into account by considering two alternative measures of the Covid-19 mortality rate (DR), namely (i) the ratio of the number of confirmed Covid-19 deaths to the total number of confirmed cases, which is widely referred to as the case-fatality rate (DR1), and (ii) the crude fatality rate (DR2), defined as the number of deaths per 100,000 of the population. Both measures are displayed in Fig. 4, whilst recorded new cases and new deaths are plotted in Fig. 5. It can be seen that DR1 increased sharply around April–May 2020 as a result of a significant rise in the number of both cases and deaths; it then kept increasing until September 2020 before falling slightly, again as a result of the evolution in the number of cases and deaths. By contrast, DR2 exhibits an upward trend throughout the sample period.

Table 1, Table 2 display the estimated coefficients in (1) under the assumption of white noise and autocorrelated errors in turn for the log regressions including DR2 as the mortality rate since DR1 was not found to be significant. Under the white noise assumption (see Table 1) the estimated value of d in the case of the S&P500 is 0.93 and is significantly below 1, which implies a small degree of mean reversion and thus is not consistent with market efficiency that requires prices to be unpredictable. The null hypothesis of I (1) cannot be rejected for USTB and ISLAM, while for GREEN the estimated d is significantly above 1; therefore market efficiency appears to hold. As for the other coefficients, the constant is significant in all four regressions; the coefficient on CHI is significant and positive in the case of USTB and that on ISP in the case of GREEN; the coefficient on DRP is always insignificant, while those on ERRF and FP are significant in all cases except for GREEN, and the coefficient on MMFPM is significant and positive for ISLAM and GREEN. Finally, the mortality rate is always significant and has a negative impact in most cases as one would expect, the only exception being the green bond market.

**Table 1:** Models for the various stock indices: estimated coefficients with white noise errors.

Regressor	Logged data			
	SP500	USTB	ISLAM	GREEN
d	0.93 (0.90, 0.95)	0.97 (0.95, 1.04)	0.96 (0.94, 1.00)	1.31 (1.21, 1.42)
Const.	8.0407 (305.83)*	5.9585 (1445.94)*	**8.3360 (396.48)**	4.6448 (991.33)*
CHI	0.0001 (0.11)	0.0004 (1.99)*	0.0003 (0.37)	0.0001 (0.65)
ISP	−0.0038 (−0.27)	−0.0011 (−0.50)	−0.0021 (−0.18)	0.0042 (1.68)*
DRP	0.0040 (0.19)	0.0007 (0.24)	0.0014 (0.09)	0.0001 (0.04)
EFFR	0.0263 (2.41)*	−0.0037 (-2.19)*	0.0199 (2.27)*	−0.0004 (−0.23)
MMFP	0.0302 (1.50)	−0.0002 (−0.06)	0.0279 (1.73)*	0.0074 (2.08)*
FP	0.0163 (1.76)*	0.0071 (2.12)*	0.0304 (1.88)*	−0.0020 (−0.57)
DR2	−1016.55 (-3.68)*	−308.82 (-5.98)*	−604.931 (-2.40)*	798.46 (3.91)*

NB: The values in parenthesis are the 95% confidence bands in the case of d whilst in the other cases they are t-values. The significant cases at the 5% level are in bold and with an asterisk.

**Table 2:** Models for the various stock indices: estimated coefficients with autocorrelated (Bloomfield) errors.

Regressor	Logged data			
	SP500	USTB	ISLAM	GREEN
d	1.18 (1.00, 1.37)	0.96 (0.94, 0.98)	1.21 (1.00, 1.41)	1.03 (0.98, 1.29)
Const.	8.0380 (301.01)*	5.9586 (1385.05)*	**8.3348 (393.24)**	4.6422 (925.71)*
CHI	−0.0007 (−0.65)	0.0003 (2.08)*	−0.0003 (−0.34)	0.0001 (0.74)
ISP	−0.0053 (−0.36)	−0.0011 (−0.48)	−0.0036 (−0.31)	0.0045 (1.65)*
DRP	−0.00007 (−0.001)	0.0011 (0.36)	0.00008 (0.05)	0.0013 (0.65)
EFFR	0.0268 (2.41)*	−0.0037 (-2.10)*	0.0195 (2.22)*	−0.0002 (−0.05)
MMFP	0.0339 (1.66)*	−0.00005 (−0.02)	0.0321 (1.98)*	0.0066 (1.72)*
FP	0.0145 (0.71)	0.0072 (2.20)*	0.0182 (1.12)	0.0004 (0.10)
DR2	1477.57 (1.87)*	−414.22 (-8.03)*	1594.84 (2.29)*	210.92 (2.58)*

NB: The values in parenthesis are the 95% confidence bands in the case of d whilst in the other cases they are t-values. The significant cases at the 5% level are in bold and with an asterisk.

Table 2 reports the results with autocorrelated errors, for which the exponential spectral model of Bloomfield [43] is used. This is a non-parametric approach as the model is only implicitly determined in terms of its spectral density function; however, it produces autocorrelations decaying exponentially as in the AR case and is stationary for the entire range of its values. Now mean reversion is only found in the case of USTB while for the other three series the estimates of d provide evidence of unit roots, which supports market efficiency. The constant is significant in all four cases, whilst the coefficient on CHI is significant only in the case of USTB, again suggesting a very limited impact of the containment measures; similarly, fiscal policy appears to be rather ineffective, as a significant impact of income support is only detected in the case of green bonds whilst debt relief has no effect in any case; again the coefficient on ERRT is significant but positive in most cases, which points to the dominance of the financial industry; the estimated coefficients for MMFPM and FP imply a wider impact of monetary announcements; finally, the coefficient on DR is significant in all four cases but is predominantly positive, which is surprising, as one would expect an exacerbation of the pandemic to depress markets.

## 4 Economic interpretation and policy implications

It is useful to provide some economic interpretation and also to reflect on the policy implications of the results presented above. Concerning the former, all the stock indices examined appear to follow a random process and thus to be unpredictable, consistently with the Efficient Market Hypothesis, which is not surprising in the case of developed markets such as the US ones. As for the impact of the policy response of the US authorities to the Covid-19 pandemic, our findings suggest that restrictions had a limited effect, since only the Treasury bond market appears to have reacted positively, and so did income support and debt relief, the former having a positive impact only in the case of green bonds whilst the latter had none. The announcements of fiscal and monetary policy support measures seem to have been more effective in lifting markets in most cases. There was also a significant impact of the effective Federal Funds rate, which is the interest rate charged to banks when they lend money to each other overnight (it is also known as the overnight rate). A rate rise is expected to decrease profitability by making debt more expensive and thus reducing the capital available for investment. As a result, in general one would expect a negative effect. However, the financial industry (banks, brokerages, mortgage companies, and insurance companies) benefits from interest rates since it can charge more for lending; therefore the estimated positive effect suggests that this sector dominates. On the whole, the most important tool at the disposal of the US policy makers appears to have been the announcement effects of both monetary and fiscal new measures (as opposed to the measures themselves); such announcements clearly had an immediate impact on agents’ expectations and thus on their investment decisions. This is an important lesson to be learned with a view to managing effectively other crises that might occur in the future.

## 5 Conclusions

This paper analyses the effects of containment measures and monetary and fiscal responses on US financial markets during the Covid-19 pandemic. More specifically, it applies fractional integration methods to analyse their impact on the daily S&P500, the US Treasury Bond Index, the S&P Green Bond Index and the Dow Jones (DJ) Islamic World Market Index over the period 1/01/2020–10/03/2021. Both the comprehensiveness of the adopted framework and the more general econometric modelling approach improve upon previous studies on this topic. In particular, we are able to shed light on whether the evolution of US stock prices has been affected by the health shock represented by the Covid-19 pandemic and the policy measures adopted in response by the US government in a transitory or permanent manner, and also on the speed of the dynamic adjustment towards the equilibrium level.

The results suggest that the four stock market indices examined are highly persistent, with orders of integration close to 1 in the majority of the cases, and mean reversion occurring only in case of the S&P500 with white noise errors and of USTB with autocorrelated ones; therefore market efficiency appears to hold in most cases. Concerning the direct impact of the pandemic, the evidence is mixed, though in most cases the mortality rate, surprisingly, appear to have affected stock and bond prices positively with autocorrelated errors. As for the effectiveness of policy responses to the pandemic, it would seem that both containment and fiscal measures had a rather limited impact, whilst there were significant announcement effects which lifted markets, especially in the case of monetary announcements. There is also evidence of a significant, positive response to changes in the effective Federal Funds rate, which suggests that the financial industry, mainly benefiting from interest rises, plays a dominant role.

The analysis of this paper can be developed in several ways. For example, the long memory approach we have used can be extended to the case where the singularity in the spectrum occurs at a frequency away from zero; by doing so, possible cycles inherent in the data can be examined. Further, the investigation can be extended to other financial assets and other countries, both developed and developing. Such issues will be examined in future papers.

### Author contribution statement

Emmanuel Abakah; Guglielmo Maria Caporale; Luis Gil-Alana: Conceived and designed the experiments; Performed the experiments; Analysed and interpreted the data; Contributed reagents, materials, analysis tools or data; Wrote the paper.

## Funding statement

This research did not receive any specific grant from funding agencies in the public, commercial, or not-for-profit sectors.

## Data availability statement

Data will be made available on request.

## Declaration of interest statement

The authors declare that they have no known competing financial interests or personal relationships that could have appeared to influence the work reported in this paper.

## References

[1] Harvey A.C. (2020).

[2] Spatt C.S. (2020). A tale of two crises: the 2008 mortgage meltdown and the 2020 COVID-19 Crisis. Rev Asset Pricing Stud.

[3] Baffes J., Nagle P. (2020). https://blogs.worldbank.org/voices/outlook-commodity-markets-and-effects-coronavirus-six-charts.

[4] Goodell J.W. (2020). COVID-19 and finance: agendas for future research. Finance Res. Lett..

[5] Ozli P.K., Arun T. (2020). https://ssrn.com/abstract=3562570.

[6] Correia S., Luck S., Verner E. (2020).

[7] Duffin E. (2020). https://www.statista.com/topics/6139/covid-19-impact-on-the-global-economy/.

[8] Baker S., Bloom N., Davis S.J., Kost K., Sammon M., Viratyosin T. (2020). The unprecedented stock market reaction to COVID-19. Covid Econ.: Vetted and R.-Time Pap..

[9] Sharif A., Aloui C., Yarovaya L. (2020). COVID-19 pandemic, oil prices, stock market, geopolitical risk and policy uncertainty nexus in the US economy: fresh evidence from the wavelet-based approach. Int. Rev. Financ. Anal..

[10] Ashraf B.N. (2020). Stock markets' reaction to COVID-19: cases or fatalities?. Res. Int. Bus. Finance.

[11] Takyi P.O., Bentum-Ennin I. (2020). The impact of COVID-19 on stock market performance in Africa: a Bayesian structural time series approach. J. Econ. Bus..

[12] Topcu M., Gulal O.S. (2020). The impact of COVID-19 on emerging stock markets. Finance Res. Lett..

[13] Tiwari A., Abakah E.J.A., Adjei R., Gil-Alana L. (2021).

[14] Insaidoo M., Arthur L., Amoako S., Andoh F.K. (2021). Stock market performance and COVID-19 pandemic: evidence from a developing economy. J. Chin. Econ. Foreign Trade Stud..

[15] Mensi W., Sensoy A., Vo X.V., Kang S.H. (2020). Impact of COVID-19 outbreak on asymmetric multifractality of gold and oil prices. Resour. Pol..

[16] Le T.L., Abakah E.J.A., Tiwari A.K. (2021). Time and frequency domain connectedness and spill-over among fintech, green bonds and cryptocurrencies in the age of the fourth industrial revolution. Technol. Forecast. Soc. Change.

[17] Umar Z., Gubareva M. (2020). A time–frequency analysis of the impact of the Covid-19 induced panic on the volatility of currency and cryptocurrency markets. J Behav Exp Financ.

[18] Bakas D., Triantafyllou A. (2020). Commodity price volatility and the economic uncertainty of pandemics. Econ. Lett..

[19] Tiwari A.K., Abakah E.J.A., Le T.L., Leyva-de la Hiz D.I. (2021). Markov-switching dependence between artificial intelligence and carbon price: the role of policy uncertainty in the era of the 4^th^ industrial revolution and the effect of COVID-19 pandemic. Technol. Forecast. Soc. Change.

[20] Ji Q., Zhang D., Zhao Y. (2020). Searching for safe-haven assets during the COVID-19 pandemic. Int. Rev. Financ. Anal..

[21] Arellano C., Bai Y., Mihalache G.P. (2020). https://www.nber.org/papers/w27275.

[22] Sène B., Mbengue M.L., Allaya M.M. (2021). Overshooting of sovereign emerging Eurobond yields in the context of COVID-19. Finance Res. Lett..

[23] Mirza N., Naqvi B., Rahat B., Rizvi S.K.A. (2020). Price reaction, volatility timing and funds' performance during Covid-19. Finance Res. Lett..

[24] Caporale G.M., Gil-Alana L., Arrese Lasaosa I. (2022). The impact of the Covid-19 pandemic on persistence in the European stock markets. Heliyon.

[25] Hale T., Petherick A., Phillips T., Webster S. (2020).

[26] Zaremba A., Aharon D.Y., Demir E., Kizys R., Zawadka D. (2021). COVID-19, government policy responses, and stock market liquidity around the world: a note. Res. Int. Bus. Finance.

[27] Wei X., Han L. (2021). The impact of COVID-19 pandemic on transmission of monetary policy to financial markets. Int. Rev. Financ. Anal..

[28] Narayan P.K., Phan D.H.B., Liu G. (2021). COVID-19 lockdowns, stimulus packages, travel bans, and stock returns. Finance Res. Lett..

[29] Zhang D., Hu M., Ji Q. (2020). Financial markets under the global pandemic of COVID-19. Finance Res. Lett..

[30] Chen W.C., Huang A.S., Chuang J.H., Chiu C.C., Kuo H.S. (2011). Social and economic impact of school closure resulting from pandemic influenza A/H1N1. J. Infect..

[31] Glantz M., Kissell R. (2013).

[32] Epstein J.M., Hammond R.A., Lempel H. (2009). https://www.brookings.edu/research/economic-cost-and-health-care-workforce-effects-of-school-closures-in-the-u-s/.

[33] Galai D., Sade O. (2006). The “ostrich effect” and the relationship between the liquidity and the yields of financial assets. J. Bus..

[34] Karlsson N., Loewenstein G., Seppi D. (2009). The ostrich effect: selective attention to information. J. Risk Uncertain..

[35] Bossmana A., Owusu P., Tiwari A.K. (2022). Dynamic connectedness and spillovers between Islamic and conventional stock markets: time- and frequency-domain approach in COVID-19 era. Heliyon.

[36] Sharma G.D., Tiwari A.K., Jain M., Yadav A., Erkut B. (2021). Unconditional and conditional analysis between covid-19 cases, temperature, exchange rate and stock markets using wavelet coherence and partial coherence approaches. Heliyon.

[37] Vo D.H., Ho C.M., Dang T.H.-N. (2022). Stock market volatility from the Covid-19 pandemic: new evidence from the Asia-Pacific region. Heliyon.

[38] Oahn T.T.K. (2022). The impact of COVID-19 vaccination on stock market: is there any difference between developed and developing countries?. Heliyon.

[39] Caporale G.M., Cerrato M. (2020). https://policyscotland.gla.ac.uk/covid-19-pandemic-and-the-economy-we-are-fighting-a-new-war/.

[40] Robinson P.M. (1994). Efficient tests of nonstationary hypotheses. J. Am. Stat. Assoc..

[41] Gil-Alana L.A., Robinson P.M. (1997). Testing of unit roots and other nonstationary hypothesis in macroeconomic time series. J. Econom..

[42] Ozkan A., Ozkan G., Yalaman A., Yildiz Y. (2021). Climate risk, culture and the Covid-19 mortality: a cross-country analysis. World Dev..

[43] Bloomfield P. (1973). An exponential model in the spectrum of a scalar time series. Biometrika.

